# Understanding factors relevant to poor sleep and coping methods in people with schizophrenia

**DOI:** 10.1186/s12888-021-03384-y

**Published:** 2021-07-26

**Authors:** Yen-Ching Chang, Megan C. Chang, Yun-Jou Chang, Ming-De Chen

**Affiliations:** 1 Department of Occupational Therapy, College of Medicine, National Cheng Kung University, 1 University Road, 70101 Tainan City, Taiwan; 2 Department of Occupational Therapy, College of Health and Human Sciences, San José State University, 1 Washington Square, San José, CA 95192-0059 USA; 3 Department of Occupational Therapy, College of Health Sciences, Kaohsiung Medical University, 100 Shin-Chuan 1st Road, Sanmin Dist, Kaohsiung City, 80708 Taiwan; 4 Department of Medical Research, Kaohsiung Medical University Hospital, 100 Tzyou 1st Road, Sanmin Dist, Kaohsiung City, 80708 Taiwan

**Keywords:** Occupation, Qualitative, Schizophrenia, Sleep, The PEO model

## Abstract

**Background:**

Sleep disruption is pervasive in people with schizophrenia, but few studies have explored their sleep experiences. This study aims to identify factors relevant to sleep problems and explore coping methods used by community-dwelling people with schizophrenia.

**Methods:**

Eighteen participants with schizophrenia were recruited from three mental health centers in Taiwan. They completed a semi-structured interview and the Pittsburgh Sleep Quality Index (PSQI) assessment. The Person-Environment-Occupation model offered a framework to assess factors related to sleep. Thematic analysis was used for the qualitative data analysis.

**Results:**

Factors related to sleep were classified under person, environment, and occupation domains. The person domain included three subthemes: psychiatric symptoms, unpleasant emotions, and frustration about sleep. The environment domain included three subthemes: sensory intrusions from the environment, quality of bedding, and roommates. The occupation domain included sleep interruption and sleep preparation. There were notable discrepancies in sleep quality between the participants’ narratives and their PSQI global scores. Regarding coping methods for poor sleep, sleep medication was the primary strategy while some participants also used other strategies, such as modifying the environment, adjusting routines, or engaging in activities that improve sleep quality.

**Conclusions:**

Psychiatric symptoms and nightmares were identified as unique sleep disruptions in people with schizophrenia, and poor economic status was also found to impact their sleep. The sleep quality of people with schizophrenia tends to be poor, as identified by the PSQI, even though they may have positive perceptions of their sleep quality. Our participants appeared to prefer to take hypnotics to address their sleep problems, which may be due to limited knowledge about alternatives. Mental health professionals are encouraged to receive training in the application of non-pharmacological approaches to support their clients’ issues related to sleep.

**Supplementary Information:**

The online version contains supplementary material available at 10.1186/s12888-021-03384-y.

## Background

Previous studies have shown that sleep disruption is pervasive in people with schizophrenia [1, 2]. Insomnia, nightmares, and circadian rhythm disruption are common in this population [3–5]. People with schizophrenia who have sleep problems tend to have poor quality of life and aggravated symptoms, such as auditory hallucinations and delusions [6, 7]. The Diagnostic and Statistical Manual of Mental Disorders (DSM-5) also suggests that in the case of individuals with mental illness and comorbid insomnia, clinicians need to address both disorders [8]. However, sleep problems in people with schizophrenia are often overlooked [9, 10]. This is possibly because sleep is not part of the diagnostic criteria for schizophrenia, unlike the criteria for anxiety and depression.

Several studies have investigated interventions intended to address sleep problems in people with schizophrenia through pharmacological and non-pharmacological approaches [11]. The most common sleep treatment is medication, but its side effects and negative impacts have been documented at length, including headaches, nausea, and lethargy [9, 12]. One of the effective non-pharmacological treatments is Cognitive Behavioral Therapy for Insomnia (CBT-I). Several studies have reported that CBT-I improves sleep quality, efficiency, and duration in people with schizophrenia [13–15]. In addition, some other non-pharmacological approaches have been used to improve sleep, including exercise, light therapy, and music therapy, but few studies have investigated the effects of such approaches on people with schizophrenia [16–18].

Individuals’ attitudes and experiences are associated with their treatment choices [19]. This is also true for people with schizophrenia, who have to manage their psychiatric symptoms along with their sleep issues. However, to date, only a limited number of studies have explored the firsthand experiences of these people with the goal of understanding the underpinning issues related to sleep [12, 20]. Without knowing their experiences and challenges, it is difficult to develop sleep interventions specifically tailored to the needs of this population. Therefore, a qualitative study is warranted to explore and understand the needs of people with schizophrenia.

In this study, we selected the Person-Environment-Occupation (PEO) model [21] as a framework to guide the data analysis. The PEO model is composed of four concepts: Person, Environment, Occupation, and Occupational Performance. *Person* refers to an individual with a set of attributes, competences, and life experiences. *Environment* covers the cultural, socio-economic, institutional, physical, and social aspects of the individual’s environment. *Occupation*, also known as tasks or activities, is defined as the activities in which a person engages during the lifetime [21]. In our study, the targeted occupation is “sleep,” which includes sleep participation and sleep preparation, based on the Occupational Therapy Practice Framework [22]. *Occupational Performance* is the outcome of the interactions between the person, environment, and occupation. The PEO model has been used to investigate the participation of various populations, such as people with physical disabilities, older adults, and people with mental illness, in various activities [23–25]. The use of the PEO model to discern the factors related to sleep problems in a comprehensive manner can assist mental health professionals with addressing the sleep problems of people with schizophrenia across person, environment, and occupation domains. Accordingly, this study aims to identify factors that influence the quality of sleep guided by the PEO model as well as explore coping methods in people with schizophrenia.

## Methods

Semi-structured interviews were used in this study to capture the sleep experiences of participants with schizophrenia. Qualitative methods are well suited for obtaining comprehensive information by which to explore the sleep issues and coping strategies of people with schizophrenia. The Pittsburgh Sleep Quality Index (PSQI) was also administered to provide a context for the sleep quality of the participants.

### Participants

Eighteen participants diagnosed with schizophrenia were recruited from three community-based mental health centers in the southern Taiwan. Generally, each center provides a weekly schedule from 9 am to 4 pm for their clients that includes such things as leisure activities and vocational training, as well as lunch and nap time. Clients live in the community and attend the activities in the center regularly during the week. The inclusion criteria were as follows: diagnosis of schizophrenia, aged 20 years or older, living in the community, past or current experiences with sleep problems, literate enough to complete a questionnaire, and willingness to share their sleep experiences via an in-person interview.

### The Pittsburgh sleep quality index (PSQI)

The PSQI, a 19-item self-report assessment, was developed to measure sleep quality in clinical populations over a 1-month time interval [26]. It includes seven component scores: 1. subjective sleep quality, 2. sleep latency (amount of time needed to fall asleep), 3. sleep duration, 4. habitual sleep efficiency, 5. sleep disturbances, 6. use of sleep medication, and 7. daytime dysfunction. The global score is a sum of the seven component scores ranging from 0 to 21, where a higher score indicates poor sleep quality. When the global score is greater than 5, one is considered to have sleep issues [26]. The PSQI has been translated into and validated in traditional Chinese [27], which is the version used in this study.

### Data collection

This study was approved by the first author’s university’s institutional review board. Data collection was conducted from July to August 2017. With the assistance of staff, the researchers visited three community-based mental health centers in southern Taiwan to recruit participants. After informed consent was obtained, each participant completed the demographic questionnaire and the PSQI assessment. In addition, they participated in a one-on-one in-person interview. The interviews were conducted in a quiet room at each of the collaborating agencies. The interview guide included questions about sleep experiences, attitudes, and coping methods for sleep problems, such as, “Under what circumstances have you encountered or noticed any sleep issues?” and “How is your sleep quality in general?” It took approximately 30 min for each interview, which was audio recorded and then transcribed verbatim. The accuracy of the transcripts was verified by a trained research assistant. Data collection continued until no new information was obtained since this indicates that the data is saturated.

### Data analysis

Thematic analysis [28] was used for the qualitative data. We analyzed the data in a deductive manner. Namely, the analysis was driven by our research inquiry: factors relevant to sleep problems and coping methods. Two researchers coded the transcripts independently using ATLAS.ti Version 8.4 (ATLAS.ti Scientific Software Development GmbH, Berlin, Germany). The initial codes were generated by reviewing the transcripts line by line, and then the codes were grouped into potential themes. Factors relevant to sleep problems were then classified based on the PEO model described in the introduction. These codes and themes were reviewed by the research team until a consensus was reached. IBM SPSS Version 20.0 (IBM SPSS Statistics for Windows, Armonk, NY: IBM Corp) was used to conduct the descriptive analysis of the demographic data and the PSQI scores.

## Results

Participant characteristics are shown in Table 1. Of the 18 participants, 50% were female; 72.2% had a high school education or less; 83.3% were single, and 55.6% participated in a sheltered employment program. The mean age was 41.4 years (range = 28–54), and the average number of years since diagnosis was 17.8 years (range = 1–29). The majority of participants (83%) had a habit of napping, possibly cultivated by the schedules provided by the community centers. The average nap time was 46 ± 25.5 min.

**Table 1:** Participant demographic characteristics (*N* = 18)

	N (%)
Age (years), mean ± SD	41.39 ± 8.16
Gender, n(%)	
Female	9(50%)
Education, n(%)	
High school graduate or below	13(72.2%)
Some college or above	5(27.8%)
Marriage, n(%)	
Single	15(83.3%)
Married	2(11.1%)
Divorced	1(5.6%)
Work, n(%)	
Full time job	1(5.6%)
Supported employment program	3(16.7%)
Sheltered employment program	10(55.6%)
Unemployment	4(22.2%)
Living environment, n(%)	
Living with family	18(100%)
Illness length (years), mean ± SD	17.83 ± 7.47
Drinking alcohol, n(%)	
Yes	0(0%)
Consuming drinks containing caffeine, n(%)	
Yes	12(66.7%)
Smoking, n(%)	
Yes	3(16.7%)
Habit of napping, n(%)	
Yes	15(83.3%)
Having a roommate or bed partner, n(%)	
Yes	6 (33.3%)

The mean PSQI global score was 9.56 (range = 3–17), and 83.3% of the participants (*n* = 15) had poor sleep quality (PSQI global score > 5). Out of the seven components scores, the top three components with higher mean scores indicating poor sleep quality were use of sleep medication (component 6), sleep latency (component 2), and daytime dysfunction (component 7; see Table 2). Almost 80% of the participants (*n* = 14) reported that they had taken medication to help them sleep during the past month, and the majority take sleep medication three or more times a week. The majority of the participants (66.7%) required 15–30 min to fall asleep (sleep latency), and some (22.2%) required between 31 and 60 min. In terms of daytime dysfunction, 44.4% reported that they have trouble staying awake one or more times a week, and 38.9% reported that it has been somewhat of a problem to keep up enough enthusiasm to get things done. Despite the aforementioned poor sleep quality identified by the PSQI global score, more than half of the participants (61.1%, 11 out of 18 participants) reported that their subjective sleep quality (component 1) was either fairly good or very good. In addition, among those reporting good sleep quality, 63.6% (7 out of 11) took sleep medication three or more times a week.

**Table 2:** The PSQI^a^ component scores and global scores

	Mean ± SD/ N(%)
1. Subjective sleep quality	1.39 ± 0.70
2. Sleep latency	1.67 ± 0.69
3. Sleep duration	0.78 ± 1.06
4. Habitual sleep efficiency	0.72 ± 1.07
5. Sleep disturbances	1.44 ± 0.70
6. Use of sleep medication	2.00 ± 1.33
7. Daytime dysfunction	1.56 ± 1.04
Global score	9.56 ± 3.73
PSQI > 5	15(83.3%)

^a^*PSQI* Pittsburgh Sleep Quality Index

The qualitative analyses documented two main themes. They were factors relevant to sleep problems and coping methods for sleep problems. An overview of the themes and subthemes is provided in the supplementary file.

### Theme 1: factors relevant to sleep problems

**Fig. 1:**
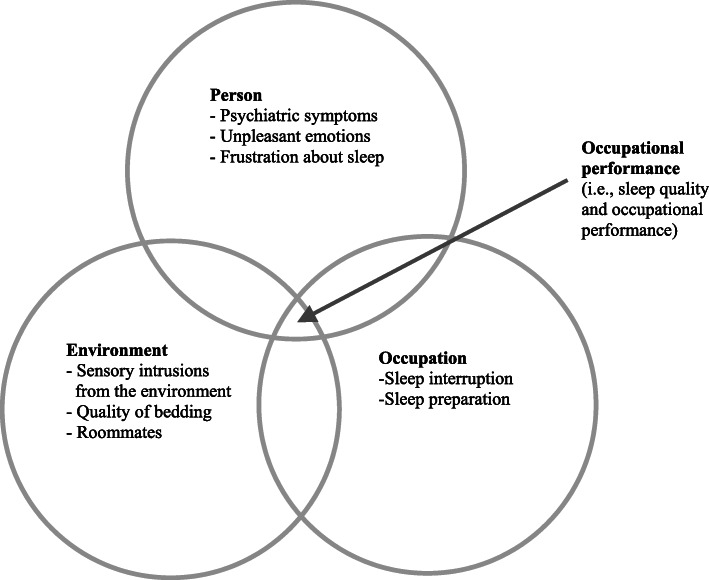
Factors relevant to sleep problems in people with schizophrenia based on the PEO model

Guided by the PEO model, we further categorized codes about factors relevant to sleep problems into three domains: person, environment, and occupation. An additional theme of occupational performance was identified to reflect the intersection of the three domains. Figure 1 shows the structure of these factors that influence sleep based on the PEO model.

### Person

The person domain included three subthemes: psychiatric symptoms, unpleasant emotions, and frustration about sleep. Psychiatric symptoms and unpleasant emotions impacted participants’ sleep quality and quantity:

“When I lie down, I hear many people talking … It feels like somebody keeps calling and chatting with me. I then wake up to eat something or go to the restroom in the middle of night” (P03).

“Some unpleasant things happened today … unsatisfactory [things]...They made me feel bad and then subsequently affected my sleep at night” (P02).

While the participants agreed that sleep is essential to functioning well during the day, some were frustrated about getting insufficient sleep:

“I think it [sleep] is important, especially for people like me who are working. The most important thing for me is to have a job, so I need to sleep. If I sleep well, I can do better at work” (P14).

“I really want to know what it feels like to have a good night sleep. I have not had enough sleep for a long time and often feel sleepy” (P04).

### Environment

In the environment domain of the PEO model, three subthemes about environmental factors that influence sleep were identified: sensory intrusions from the environment, quality of bedding, and roommates. Sensory intrusions included lighting, ambient sound, and temperature. The participants frequently preferred various types of lighting and sound ambience for sleep. Some people liked darkness or quiet, while others preferred leaving on a night lamp or radio to cope with symptoms:

“I turn on a nightlight because I feel insecure when it is too dark” (P06).

“When I sleep, I need to turn on the radio. If I don’t do this, I will have a dream, and the dream is usually pretty weird” (P14).

Room temperature was repeatedly mentioned in the interviews. Participants stated that when the room temperature is high, it is challenging to fall asleep. Some mentioned limited resources available for adjusting the temperature: “My mom is frugal. She asks us not to turn on the air conditioner and to only turn on the fan” (P04). The participants also shared their concerns about bedding and roommates. Some described problems with their mattress (too small or too old), whereas some stated that their sibling’s sleep habits or snoring disrupted their sleep.

“I sleep on a twin size mattress that’s quite shabby. I would like to get a new and bigger one, so I would have a bigger and better space for sleep” (P14).

### Occupation

The occupation domain was composed of two subthemes: sleep interruption and sleep preparation. Many participants reported that they woke up due to nightmares or the need to urinate. Such interruptions disrupted their quality of sleep:

“Sometimes I have a nightmare and then rouse suddenly from sleep” (P07).

“If I drink too much water, I cannot fall asleep. If I wake up to go to the restroom, I cannot fall back to sleep easily” (P16).

As for sleep preparation, many participants were aware of how activities they participate in may impact their sleep:

“As long as I don’t drink tea and don’t have any conflicts or bad situations during my work or before 11:30 p.m., it will be all right” (P14).

“If I don’t use my smartphone [before sleep], it [my sleep] will be fine” (P18).

### Sleep quality and occupational performance

Occupational performance is the core of the PEO model, which is the center of the Venn diagram in Fig. 1. It is complex and is shaped by the person, environment, and occupation domains. Law et al. (1996) stated that the subjective quality of occupational performance can be measured by self-reporting, which is self-reported sleep quality in this study. In the interviews, many participants were somewhat satisfied with their sleep quality, which was consistent with their responses to the first PSQI component about their subjective sleep quality. Some participants said, “If I take medicine [hypnotics], I am satisfied. However, if I don’t take medicine, which I have tried, I will wake up and cannot sleep through the night” (P10). “As long as I take sleep medication before 11 p.m. and keep my emotions stable, I am satisfied with my sleep quality” (P14). Some participants perceived that as long as they are able to fall asleep, they feel good about sleep quality: “I feel good [about sleep quality] because I can fall asleep. I think being able to fall asleep is the key to a good night’s sleep” (P02).

### Theme 2: coping methods for sleep problems

The participants described several coping methods they have applied to resolve sleep issues, including sleep medication and non-pharmacological strategies. When we inquired how they managed their sleep problems, we discovered that all of the participants consulted their psychiatrists about sleep problems. However, some consulted psychiatrists only, while others also sought suggestions from other mental health professionals and/or their significant others. Additionally, the participants stated that suggestions or assistance received from psychiatrists and professionals were mostly related to medication management, noting that a few suggested exercise or attending a mental health center to develop routines that would improve their sleep.

### Sleep medication

Almost all participants relied on sleep medication as the primary strategy for sleep problems and could name the hypnotics they take. Two subthemes were included: experiences with taking sleep medication and willingness to adopt alternative treatments. Some participants were used to taking sleep medication, but some were unsatisfied with the side effects. Types of concerns about side effects were exemplified by the following observations: “I don’t like it [taking sleep medication] because the side effects are too strong to be functional the next day. I feel like a zombie or a walking corpse and feel dizzy” (P04). “After taking sleep medication, I will feel really bad the next day. The effect of the medicine seems to be lasting longer. I just take the medication [hypnotics] before bed. Sometimes, I still feel sleepy when I wake up” (P06).

When probing further about alternative treatments intended to reduce reliance on sleep medication, many participants expressed reluctance:

“I do not want to try an alternative treatment. I think it is better to follow the psychiatrist’s prescriptions” (P02).

“I have tried it [reducing the dosage of the sleep medication]. I’ve tried it two or three times. It did not work. I still need to rely on it. My psychiatrist said to me that it is ok to take hypnotics. Because I have this disease, I need to take medication before bed” (P11).

### Non-pharmacological strategies

In addition to sleep medication, some participants described combining it with other methods to help them sleep, including modifying the environment, adjusting routines, and engaging in activities that improve sleep quality. Many participants mentioned adjusting the room temperature: “If it is hot, I will turn on the fan” (P12). Some adjusted their time to go to bed: “Previously, I didn’t go to bed early. I went to bed at 10 or 11 pm. Now, I usually go to bed at 9 pm because I think I should not go to bed late if I want to have better sleep quality” (P02). In addition, many also chose to engage in various preferred activities to improve their sleep:

“If I read the Bible and pray before sleep, I will sleep better and seldom have a dream that night” (P16).

“[I] listen to music. Sometimes, I will do some stretching to make me tired … it is easier to fall asleep this way” (P06).

## Discussion

To our knowledge, this is the first study to explore sleep problems in people with schizophrenia using the PEO model. In addition, this study was conducted in Taiwan, and the results can provide perspectives of people with schizophrenia within a non-Western context. We identified two major themes: factors relevant to sleep problems and coping methods for sleep problems. People with schizophrenia had unique concerns related to sleep in the person, environment, and occupation domains. Discrepancies between subjective sleep quality and the PSQI global score were found. Additionally, the participants used sleep medication as the primary strategy for sleep problems, while some participants used additional strategies to improve their sleep.

Based on the PEO model, factors relevant to sleep problems were categorized into three domains. In the person domain, psychiatric symptoms emerged as the dominant concerns because participants could not sleep well due to active symptoms, especially hallucinations. In the environment domain, room temperature was the most common theme. This may be because it was summer at the time of the interviews, and air conditioners were not accessible for many participants living in Southern Taiwan, which is located in the tropics. As expected, it is really uncomfortable to sleep without air conditioning in extremely warm environments. Since people with schizophrenia typically have low incomes [29], they might not be able to purchase air conditioners/heaters, own a good bed, or may not even be able to sleep alone. Our results echo previous recommendations suggesting that more adjustments to the sleep environment of people with schizophrenia are often needed [10]. In the occupation domain, many participants suffered from sleep interruptions because of nightmares or the urge to urinate. They also noted some behaviors should be avoided to have better sleep, such as drinking tea. In addition, the relationships between sleep and related factors are complicated [9, 30] because they involve interactions between person, environment, and occupation. For example, some participants leave the light on in order to cope with their sense of insecurity. Findings have shown that feelings of insecurity (Person) influence modifications to one’s environment (Environment). However, these environmental modifications are not typically recommended by sleep hygiene educational practitioners since such strategies may further impact sleep performance. Generally, people with schizophrenia identify factors related to sleep problems that are similar to those reported by people with insomnia [31, 32]. However, psychiatric symptoms and frequent nightmares are unique concerns of people with schizophrenia [33], and having limited resources to adjust the environment in order to improve sleep is also a specific challenge for this population. Future sleep interventions for people with schizophrenia need to consider these issues and provide appropriate assistance.

Many of the participants noted that their sleep quality was acceptable in both their narratives and responses to component 1 (subjective sleep quality) of the PSQI. However, over 80% of participants appeared to have poor sleep quality based on the PSQI global score. Of the seven components of the PSQI, use of sleep medication was the component with the highest score, and among those reporting fairly good or very good sleep quality, 63.6% (7 out of 11) took hypnotics three or more times a week. Unlike people with insomnia, who tend to take fewer sleep medications [34], in this study, people with schizophrenia who also had sleep issues had a higher level of acceptance of sleep medications, which is possibly due to their long history of taking antipsychotic medications. The qualitative data revealed that people with schizophrenia may accept a status quo of sleep quality as long as they are able to fall asleep even if it requires taking hypnotics. Such discrepancies between perceptions of overall sleep quality and the PSQI global score were also found in a recent study [35]. Hence, it is important to note that positive perceptions of sleep quality should not be considered as not having sleep problems in people with schizophrenia. Instead, their sleep quality tends to be poor as determined by a multi-component sleep quality assessment (i.e., the PSQI).

Most participants relied on medication to resolve their sleep problems, and some stated that they were dissatisfied with the side effects of medication, which made them tired and dizzy. This result was consistent with the findings of previous studies [9, 36]. However, we found that most of the participants appeared to prefer to take sleep medication to relieve their sleep problems. Although they disliked the side effects, most of them chose to maintain the status quo because they were afraid of suffering worse sleep quality after trying a new method or reducing their dosage. They were willing to use other strategies along with sleep medications, but they seemed to resist reducing their reliance on sleep medications. Previous negative experiences with changing or reducing medications, either anti-psychotics or hypnotics, may explain this, at least in part. Additionally, studies have found that people with mental illness lack knowledge about sleep and alternative treatments [30, 37], which may also influence their motivation to receive or engage in other coping methods.

In addition to sleep medications, several participants applied various non-pharmacological coping strategies to mitigate their sleep problems. These coping methods typically targeted the environment and occupation domains of the PEO model. Environmental adjustments may be implemented instantly (e.g., turning on an air conditioner and replacing an old mattress). However, as we discussed above, people with schizophrenia may not have sufficient financial support to do these things. As for strategies for the occupation domain, such as adjusting routines and engaging in self-selected activities, they yield only gradual effects related to improving sleep [9] since behavioral change takes time. To prepare for a good night’s sleep, the participants in this study attempted to modify their routine or form habits (e.g., regular exercise and pre-sleep Bible reading). Such changes require inner drive and may require continuous support and assistance. Thus, mental health professionals are encouraged to help clients develop daily routines by incorporating certain activities that are not only meaningful to them but also beneficial to the sleep process.

It is interesting to note that while all of the participants consulted their psychiatrists about sleep problems, some participants did not seek suggestions from other mental health professionals. Additionally, suggestions from psychiatrists and other mental health professionals were mostly related to medication. This may echo findings from the previous studies indicating that healthcare providers have limited training and knowledge related to sleep issues [30, 38]. Current clinical guidelines recommend Cognitive Behavioral Therapy for Insomnia (CBT-I) as the first line of treatment for insomnia [39, 40]. However, it should be noted that no participants in our study mentioned they received CBT-I, which is consistent with the findings from other studies [9, 41]. These findings suggest that CBT-I is not yet commonly provided for community-dwelling people with schizophrenia who have sleep issues. Thus, it is important for mental health professionals to receive training in CBT-I and/or other non-pharmacological approaches that meet the needs of people with schizophrenia [10, 42].

This study has several limitations. First, 18 participants were recruited from three community mental health centers in Taiwan. This may limit the transferability of the findings to those who are illiterate, located in different geographical regions, have different cultural backgrounds, or those who do not receive community-based mental health services. Nevertheless, the study added preliminary insights into this under-investigated field. Second, information about the participants’ psychopathology, medications, and BMI was not collected. This may limit the understanding of the characteristics of participants related to their sleep problems. Third, the transcripts were not reviewed and checked by the participants. However, the interviewers made an effort to confirm the responses with the participants during the interview. Additionally, two researchers coded the data independently and discussed the data with the research team in order to reduce potential bias throughout the analysis process.

## Conclusions

The current study provides preliminary information that will lead to a better understanding of the sleep experiences of people with schizophrenia. The PEO model provided a comprehensive framework by which to assess factors relevant to sleep problems. Psychiatric symptoms and nightmares comprised the unique sleep disruptions in this population, and poor economic conditions related to the environment domain also impacted their sleep participation. In addition, mental health professionals need to be aware that the sleep quality of people with schizophrenia tends to be poor, as identified by the PSQI, even though they may have positive perceptions of their sleep quality. Furthermore, our participants appeared to prefer to take hypnotics to address their sleep problems, which may be due to limited knowledge about alternatives. Mental health professionals are encouraged to receive training on non-pharmacological interventions (e.g., CBT-I) to support sleep issues experienced by people with schizophrenia. Therefore, people with schizophrenia can access various interventions tailored to their needs to improve their sleep problems.

## Supplementary Information

**Additional file 1.** An overview of themes and subthemes.

## Data Availability

The datasets generated and/or analyzed during the current study are not publicly available due to the protection of participant privacy but are available from the corresponding author on reasonable request.
