# Supplementary material for: Understanding factors relevant to poor sleep and coping methods in people with schizophrenia
Source: BMC Psychiatry. 2021 Jul 26;21:373. doi: 10.1186/s12888-021-03384-y (PMC8311940; doi:10.1186/s12888-021-03384-y)
Supplement: Supplementary file 1 — Additional file 1. An overview of themes and subthemes. [file 12888_2021_3384_MOESM1_ESM.docx]

An overview of themes and subthemes

| Themes/Subthemes | | Examples of quotes |
| --- | --- | --- |
| 1. Factors relevant to sleep problems | |  |
|  | **Person** |  |
|  | Psychiatric symptoms | *“When I lie down, I hear many people talking…It feels like somebody keeps calling and chatting with me. I then wake up to eat something or go to the restroom in the middle of night” (P03).* |
|  | Unpleasant emotions | *“Some unpleasant things happened today … unsatisfactory [things]...They made me feel bad and then subsequently affected my sleep at night” (P02).* |
|  | Frustration about sleep | *“I really want to know what it feels like to have a good night sleep. I have not had enough sleep for a long time and often feel sleepy” (P04).* |
|  | **Environment** |  |
|  | Sensory intrusions from the environment | *“I turn on a nightlight because I feel insecure when it is too dark” (P06).* |
|  | Quality of bedding | *“I sleep on a twin size mattress that’s quite shabby. I would like to get a new and bigger one, so I would have a bigger and better space for sleep” (P14).* |
|  | Roommates | *“She [my sister] influences my sleep. I need to go to sleep earlier than her. Otherwise, she snores sometimes” (P01).* |
|  | **Occupation** |  |
|  | Sleep interruption | *“Sometimes I have a nightmare and then rouse suddenly from sleep” (P07).* |
|  | Sleep preparation | *“If I don’t use my smartphone [before sleep], it [my sleep] will be fine” (P18).* |
|  | **Sleep quality and occupational performance** | *“If I take medicine [hypnotics], I am satisfied. However, if I don’t take medicine, which I have tried, I will wake up and cannot sleep through the night” (P10).* |
| 1. Coping methods for sleep problems | |  |
|  | **Sleep medication** |  |
|  | Experiences with taking sleep medication | *“I don’t like it [taking sleep medication] because the side effects are too strong to be functional the next day. I feel like a zombie or a walking corpse and feel dizzy” (P04).* |
|  | Willingness to adopt alternative treatments | *“I do not want to try an alternative treatment. I think it is better to follow the psychiatrist’s prescriptions” (P02).* |
|  | **Non-pharmacological strategies** |  |
|  | modifying the environment | *“If it is hot, I will turn on the fan” (P12).* |
|  | Adjusting routines | *“Previously, I didn’t go to bed early. I went to bed at 10 or 11 pm. Now, I usually go to bed at 9 pm because I think I should not go to bed late if I want to have better sleep quality” (P02).* |
|  | Engaging in activities that improve sleep quality | *“[I] listen to music. Sometimes, I will do some stretching to make me tired… it is easier to fall asleep this way” (P06).* |
